# Airway management for a patient with tracheobronchomegaly undergoing lobectomy: a case report

**DOI:** 10.1186/s12871-023-02324-5

**Published:** 2023-11-02

**Authors:** Sai-Nan Wang, An-Shi Wu, Jin-Bai Miao, Shuo Chen, Jia Jiang

**Affiliations:** 1 Department of Anesthesiology, Beijing Chao-yang Hospital, Capital Medical University, Gongtinanlu 8#, Chaoyang, Beijing, 10020 China; 2 Department of Thoracic surgery, Beijing Chao-yang Hospital, Capital Medical University, Beijing, China

**Keywords:** Airway management, Double-lumen Foley catheter, Laryngeal mask airway, One-lung ventilation, Tracheobronchomegaly

## Abstract

**Background:**

Tracheobronchomegaly (TBM) is a rare disorder mainly characterized by dilatation and malacia of the trachea and major bronchi with diverticularization. This will be a great challenge for airway management, especially in thoracic surgery requiring one-lung ventilation. Using a laryngeal mask airway and a modified double-lumen Foley catheter (DFC) as a “blocker” may achieve one-lung ventilation. This is the first report introducing this method in a patient with TBM.

**Case presentation:**

We present a 64-year-old man with TBM receiving left lower lobectomy. Preoperative chest computed tomography demonstrated a prominent tracheobronchial dilation and deformation with multiple diverticularization. The most commonly used double-lumen tube or bronchial blocker could not match the distorted airways. After general anesthesia induction, a 4# laryngeal mask was inserted, through which the modified DFC was positioned in the left main bronchus with the guidance of a fiberoptic bronchoscope. The DFC balloon was inflated with 10 ml air and lung isolation was achieved without any significant air leak during one-lung or two-lung ventilation. However, the collapse of the non-dependent lung was delayed and finally achieved by low-pressure artificial pneumothorax. The surgery was successful and the patient was extubated soon after the surgery.

**Conclusions:**

Using a laryngeal mask airway with a modified double-lumen Foley catheter acted as a bronchial blocker could be an alternative method to achieve lung isolation.

**Supplementary Information:**

The online version contains supplementary material available at 10.1186/s12871-023-02324-5.

## Background

Tracheobronchomegaly (TBM) first described by Mounier Kuhn in 1932 [1] is a very rare disorder characterized by marked tracheobronchial dilation and flaccidity with diverticularization from the severe atrophy of elastic fibers with thinning of the muscularis mucosa in the central airway [2–4] .The airway management of a patient with TBM can be challenging and more complex for one-lung ventilation (OLV). To the authors’ knowledge, very few cases with TBM receiving OLV were reported [5–8]. Here, the authors presented a patient with TBM receiving left lower lobectomy. The most commonly used double-lumen tube (DLT) or bronchial blocker (BB) cannot match the dilated airways. The authors thus used a laryngeal mask airway (LMA) to guarantee the ventilation with a modified double-lumen Foley catheter (DFC) acted as a BB to accomplish the lung isolation.

The patient’s written informed consent was obtained.

## Case presentation

A 64-year-old man (BMI of 21.5 kg/cm^2^) was scheduled for left lower lobectomy due to lung cancer. The patient complained of chest tightness with pinprick pain in the left lower chest. He had no cough, expectoration, or dyspnea before surgery. Preoperative arterial blood gas on air was normal and the pulmonary function test suggested a mild obstructive pulmonary disorder.

**Fig. 1:**
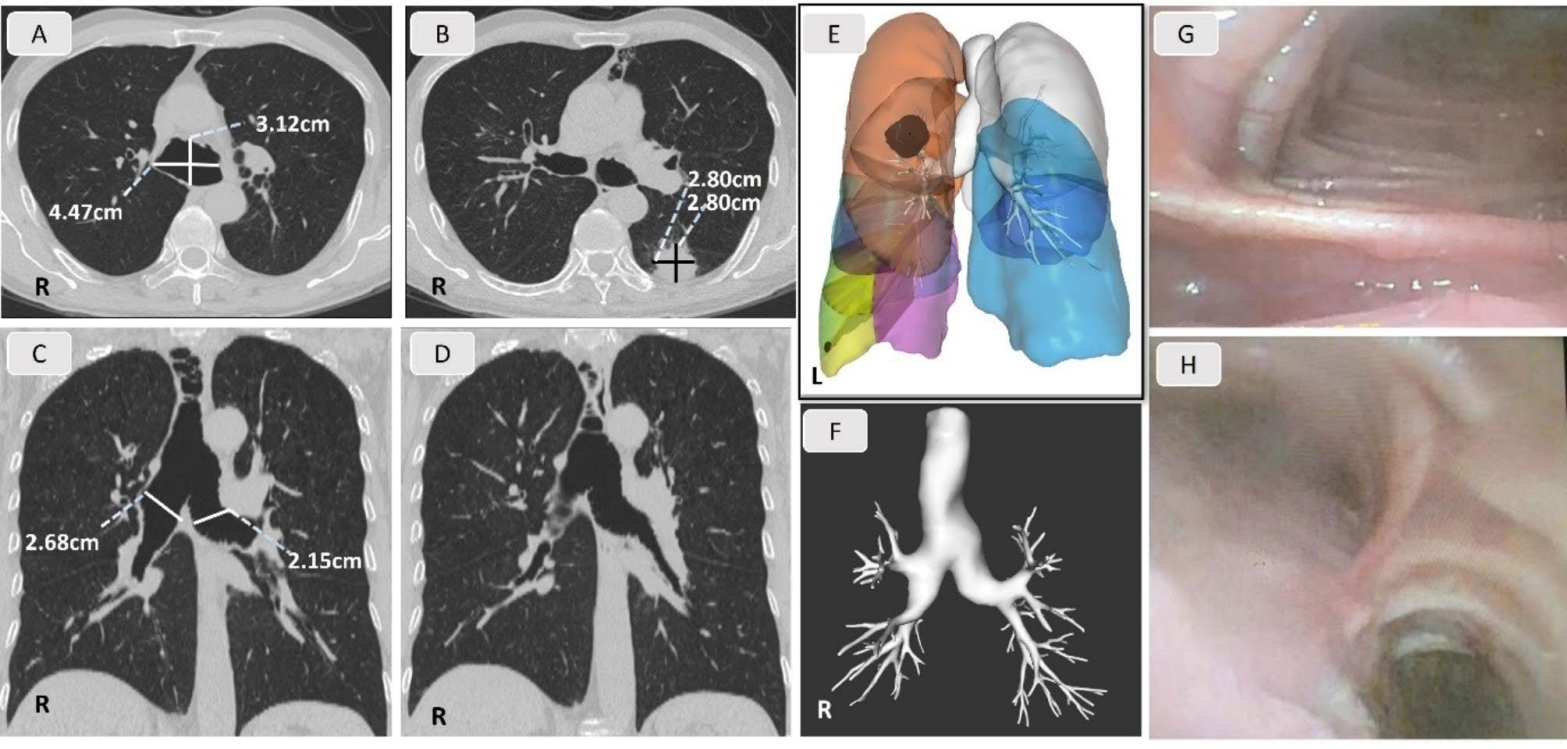
Preoperative imaging and fiberoscopic findings. **a-b**. The transverse thoracic CT. The maximum anteroposterior and axis diameters of the trachea and the tumor. **c-d**. The coronal thoracic CT. The maximum diameters of the left and right main bronchus and the deformed central airway with multiple diverticula. **e-f**. Coronal thoracic CT reconstruction. The tumor and the distorted airway. **g-h**. Fiberoptic bronchoscopy. The trachea and the major bronchi at the carina

Preoperative chest computed tomography (CT) and coronal CT reconstruction demonstrated a mass in the left lower lobe with a diameter of approximately 2.8 centimeter and invading the pleura (Fig. 1B and E). Besides, a prominent tracheobronchial dilation and deformation with multiple diverticularization were shown (Fig. 1A F). On transverse image, the maximum anteroposterior and axis diameters of the trachea were 3.12 and 4.47 cm respectively (Fig. 1A). On coronal images, the left and right main bronchus were 2.15 and 2.68 cm at their maximum diameters respectively (Fig. 1C). Preoperative fiberoptic bronchoscopy further showed the irregular ellipse shape of the central airway with thinning muscularis mucosa and prominent diverticularization (Fig. 1G H). A diagnosis of TBM was thus made [9].

**Fig. 2:**
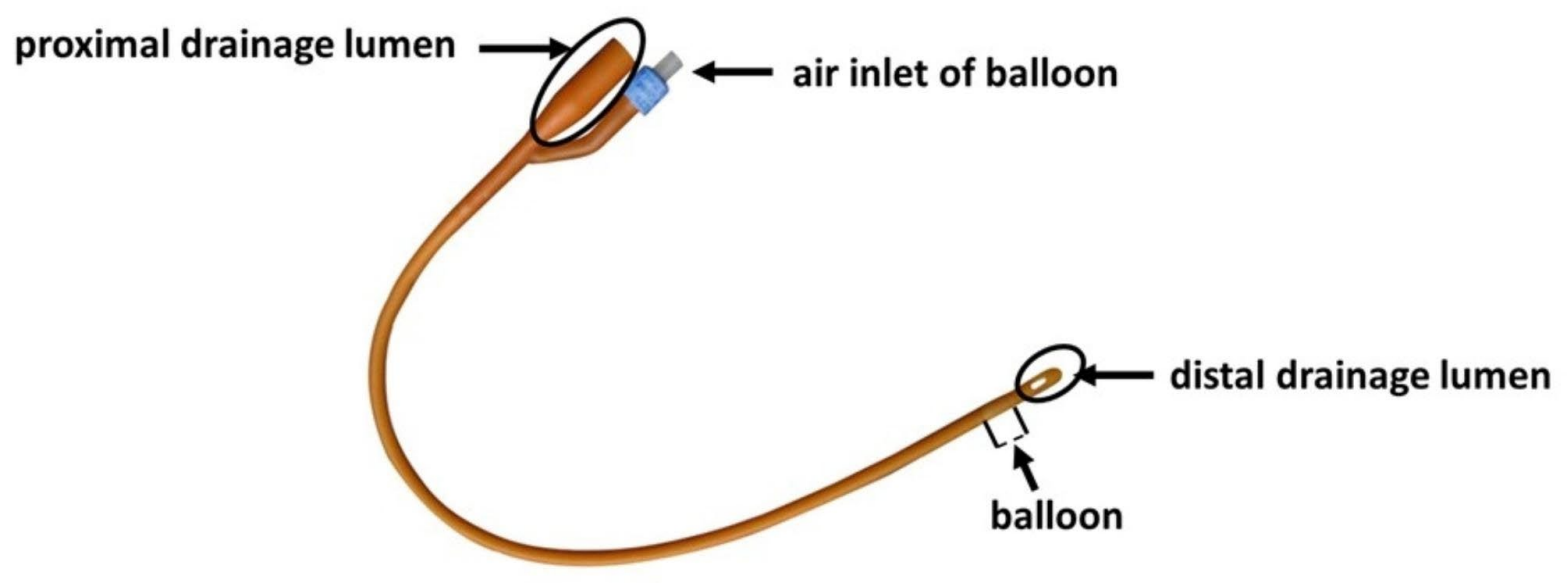
The modification of a double-lumen Foley catheter. The parts enclosed by the ovals were the parts cut off

**Fig. 3:**
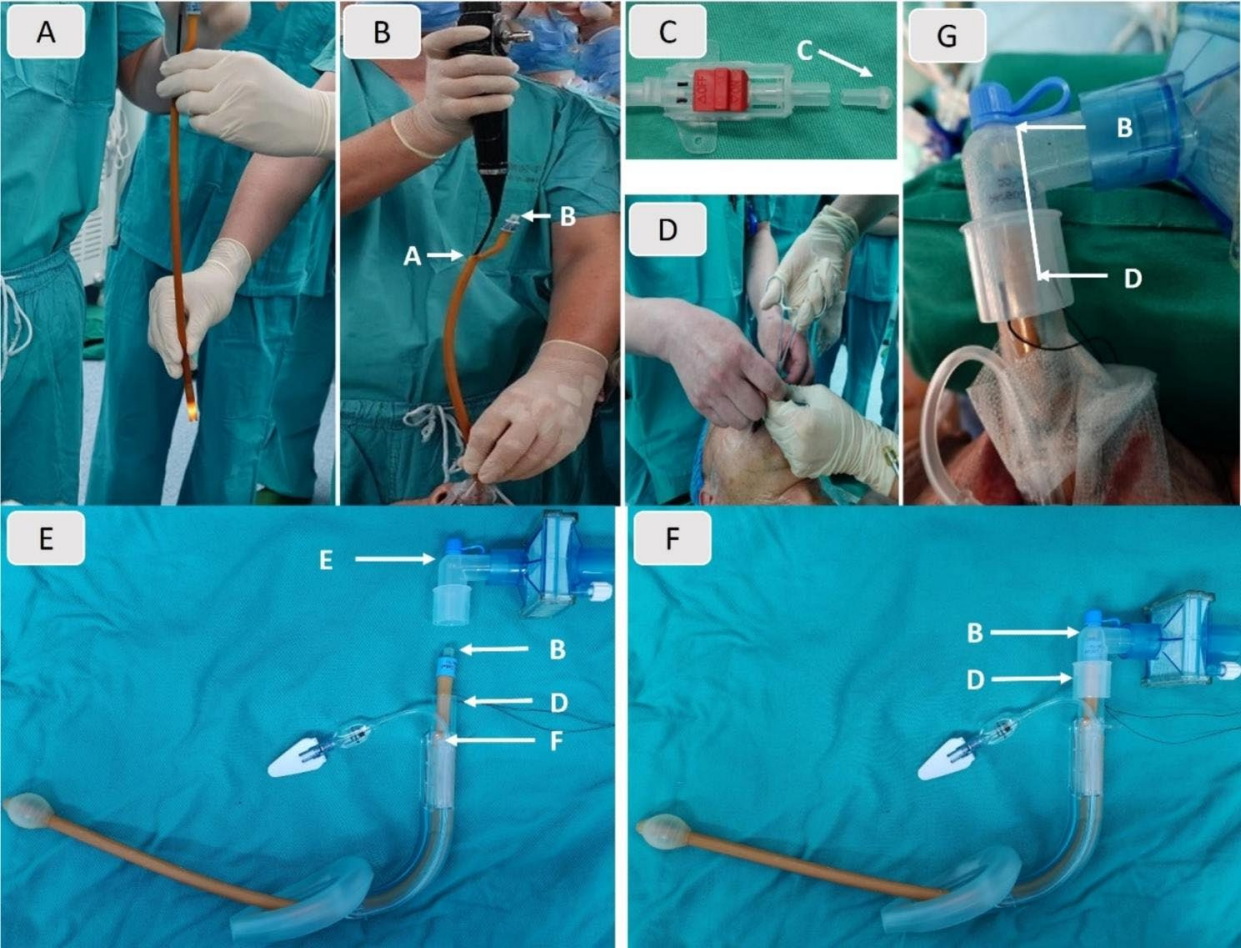
The procedure for lung isolation. **a**. Measuring the DFC’s length and the distance from the left main bronchus to the proximal opening of the LMA. **b**. Inserting the modified DFC into the left main bronchus. The arrow “A” represented the proximal opening of the modified DFC’ drainage lumen, the arrow “B” represented the proximal end of DFC, which was also the air inlet of the DFC’ balloon. **c-d**. The proximal opening of the modified DFC’ drainage lumen (arrow “A”) was occluded with a sealing cap of a 20 G BD arterial cannula (arrow “C”) to prevent deflating (Becton Dickinson Infusion Therapy Systems Inc., Sandy, Utah, USA). **e**. Status before the breathing circuit and the LMA was connected. The arrow “D” represented the proximal opening of the LMA, the arrow “E” represented the corner of the breathing circuit, the arrow “F” represented the oesophageal drain tube. **f-g**. Connecting the breathing circuit and the LMA together. The part of the DFC that longer than the distance from the left main bronchus to the proximal opening of the LMA was the distance from arrow “B” to arrow “D”, which was about 3 centimeters so that the proximal end of the DFC (arrow “B”) could be fixed in the corner of the breathing circuit (arrow “E”)

Considering that a traditional DLT or BB might have a significant peri-tubal air leakage and an arbitrary intubation might damage the airway, a method by using a LMA with a homemade BB was tried. The homemade BB was modified by a DFC with the distal drainage lumen cut to allow the fiberoptic bronchoscope (FOB) pass and the proximal drainage lumen cut to fit in the LMA (Fig. 2). In order to minimize the airway resistance, the size of the DFC should be the smallest one that can pass through a given LMA and allow the FOB to pass through. The DFC’s length should be about 3 centimeters longer than the distance from the left main bronchus to the proximal opening of the LMA (Fig. 3E-G). The volume of air inflated into the balloon should be predetermined to just fit the dilated left main bronchus. After testing in two patients with normal airway receiving OLV successfully, a 4# LMA (Haopu Biotechnology Co. Ltd., Hangzhou, Zhejiang, China) and a 24^#^ DFC (Bard Sdn. Bhd., Kulim, Kedah, Malaysia) were chosen for this patient.

On entering the operation room, the ECG, SpO_2_, noninvasive blood pressure, and arterial blood pressure were monitored. General anesthesia was induced with 1 mg midazolam, 15 µg sufentanil, 80 mg propofol, and 60 mg rocuronium. After losing the consciousness, the patient was intubated with a 4^#^ LMA, through which the modified DFC was positioned in the left main bronchus with the guidance of a FOB (Fig. 3A and B). The DFC balloon was inflated with 10 ml air and the proximal opening of the drainage lumen was occluded with a sealing cap of a 20 G BD arterial cannula to prevent deflating (Becton Dickinson Infusion Therapy Systems Inc., Sandy, Utah, USA) (Fig. 3C and D). The effect of lung isolation was confirmed by auscultation. No significant air leak occurred during one-lung or two-lung ventilation. During OLV, the surgeons confirmed lung isolation by witnessing the non-dependent lung had no apparent movement with ventilation and by touching the DFC balloon through the outer wall of left main bronchus. However, the collapse of the non-dependent lung was delayed even with the disconnection of the LMA and the breathing circuit. Artificial pneumothorax with lower pressure was then used to accelerate the lung collapse only at the beginning of the surgery. When lung re-expansion was required, the balloon needed to be evacuated, which further confirmed the correct position of the modified “blocker”. The two-lung ventilation resumed after the DFC was withdrawn. Fiberoptic bronchoscopy was performed again to exclude any potential airway injury. The surgery lasted for 3 h and 40 min. The surgeons were satisfied with the airway management. Immediately after the surgery, the patient’s conscious and breath recovered and the LMA was removed. One week after the surgery, the patient was discharged without any complications.

## Discussion and conclusion

This is the first report of using a LMA and a modified Foley catheter as a “blocker” to achieve OLV in a patient with TBM. For anesthesiologists, the biggest challenge is the OLV strategy meanwhile minimizing the airway injury.

The four commonly used methods for achieving OLV are DLT, BB, inserting an endotracheal tube into main bronchus, or artificial pneumothorax. For this case, each has their own defects. The most frequently used DLT or its alternative - an endotracheal tube with a BB might fail to occlude the main trachea and left bronchus even the largest size (41 F) available in our center was chosen. Besides, even under the guidance of a fiberscope, the endotracheal tube or a DLT tends to damage the fragile tracheobronchial wall, or strays into diverticulum leading to perforation. To date, there has been no report of successful lung isolation with DLT or BB in patients with prominent TBM. Another strategy to provide OLV is inserting an endotracheal tube (ETT) into the dependent bronchus. Considering that the low-pressure cuff has a lower risk of mucosal injury even applying increased volume of air to the cuff, this seems to be a better choice for patients with TBM [7, 8]; however, it should be noted that for a patient with TBM, the cuff site will be very inclined to be dilated and subsequently narrowed due to the fibrous tissue formation. It was thus suggested that intubation the trachea with an uncuffed tube together with throat packing to reduce the risk of gas leakage and aspiration [5]. Artificial pneumothorax will be the last choice when the lung isolation is not satisfying, but a high-pressure artificial pneumothorax has an adverse effect on hemodynamics and may result in hypoxia and hypercapnia. A supraglottic device can avoid peri-tubal air leakage and the airway damage, therefore, LMA was considered preferable for this patient.

To achieve OLV, the authors chose to modify a DFC for several reasons. First and foremost, the balloon of a 24^#^ DFC is up to 32 mm in diameter and the diameter is 30 mm after inflating 10ml gas or water, which is within the manufacturer’s recommended range. The volume of inflated air must be predetermined to just fit the dilated main bronchus to prevent the airway damage. Second, the texture of the DFC is relatively soft, the distal end is not as sharp as an ETT or DLT, the damage to the airway is thus limited. Although inserting the ETT into the dependent bronchus may achieve lung isolation, the patient might be more susceptible to airway damage due to the more pronounced diverticula in his right bronchus. Third, the modified DFC can pass through the LMA and through which a fiberoptic bronchoscope can pass. Certainly, in order to reduce airway resistance, the largest acceptable LMA and the smallest DFC should be selected.

In recent years, non-intubated thoracic surgery (NIVATS) has been on the rise with the advantages of no damage to the airway and fast recovery. This method requires the technique and cooperation of both anesthesiologists and surgeons. In our center, NIVATS has recently been applied only in technically simple VATS such as wedge resection, which means our technique is far from maturity. In addition, the surgery was expected to be complex for the location, size, and invasion of the pleura of the tumor. As indicated previously, technically difficulty and long procedures warranted a strong recommendation to avoid non-intubated procedure. Moreover, a key issue in NIVATS is the need to minimize the cough reflex [10]. In patients with tracheobronchomegaly, the airway is deemed to be sensitive and the cough reflex will be more likely to be triggered. For all these reasons above, the NIVATS technique was not adopted in this patient.

There are some limitations of this modified “bronchial blocker”. First, although the DFC balloon successfully prevented ventilation of the non-dependent lung, the lung collapse was not very satisfactory. Considering the incidental balloon displacement and the difficulty of repositioning due to the pathophysiological changes, the balloon was not deflated at the beginning of OLV, which might delay the lung collapse. Second, a small amount of air leakage around the balloon was unavoidable due to the mismatching of a spherical DFC balloon with the distorted bronchi. In addition, advancing the catheter to the suitable position was time-consuming which requires accurate preoperative measurements and experienced bronchoscopy. Finally, it was not convenient for effective sputum aspiration.

In summary, airway management for a patient with TBM is challenging, especially when OLV is needed. How to achieve lung isolation and minimize the airway damage for such an enlarged distorted fragile tracheobronchial system is the core problem for anesthesiologists. Using a laryngeal mask airway with a modified double-lumen Foley catheter acted as a bronchial blocker could be an alternative method to achieve lung isolation.

### Electronic supplementary material

Below is the link to the electronic supplementary material.

Supplementary Material 1

## Data Availability

The raw data supporting the conclusions of this article will be made available by the author Sai-Nan Wang (E-mail: sainanwang@mail.ccmu.edu.cn), without undue reservation.

## Abbreviations

TBM: Tracheobronchomegaly
DFC: Double-lumen Foley catheter
OLV: One-lung ventilation
DLT: Double-lumen tube
BB: Bronchial blocker
LMA: Laryngeal mask airway
CT: Computed tomography
FOB: Fiberoptic bronchoscope
ETT: Endotracheal tube
NIVATS: Non-intubated thoracic surgery

## References

[1] Mounier-Kuhn P (1932). Dilatation De La Trachee: constatations radiographiques et bronchoscopiques. Lyon Med.

[2] Bousnina S, Smaoui M, Hassine E, Marniche K, Fekih LE, Megdiche ML, Chabbou A (2011). Mounier-Kuhn syndrome: a rare cause of bronchial dilation. Tex Heart Inst J.

[3] Pacheco GG, Jones AM, Yao J, Kleiner DE, Taveira- Da Silva AM, Moss J (2018). Mounier-Kuhn Syndrome Mimicking Lymphangioleiomyomatosis. Chest.

[4] Krustins E, Kravale Z, Buls A (2013). Mounier-Kuhn syndrome or congenital tracheobronchomegaly: a literature review. Respir Med.

[5] F. M, MESSAHEL. Tracheal dilatation followed by stenosis in Mounier–Kuhn syndrome: a case report. Anaesthesia. 1989; 44:227–9. 10.1111/j.1365-2044.1989.tb11229.x 2705609

[6] Maxwell MJ, Pollock JG, Iftikhar SY, Chesshire NJ (2009). One-lung ventilation in a patient with tracheobronchomegaly: a case report and literature review. Eur J Anaesthesiol.

[7] Subramani S, Freeman B, Rajagopal S (2015). Anesthetic considerations for bilateral lung transplantation in Mounier-Kuhn Syndrome. J Cardiothorac Vascular Anesth.

[8] Zhang T, Wang Q, Zhou B (2021). Intraoperative airway management in a patient with tracheobronchomegaly: a case report. J Clin Anesthesiology.

[9] Marom EM, Goodman PC, Mcadams HP (2001). Diffuse abnormalities of the trachea and main bronchi. Ajr Am J Roentgenol.

[10] Anile M, Vannucci J, Ferrante F, Bruno K, De Paolo D, Bassi M, Pugliese F, Venuta F, tNIG, Diso D, Poggi C, Pecoraro Y, Carillo C, Amore D, Mottola E, Bianco M, Centofanti A, Vaz Sousa RF, Sebastianelli V, Pio Evangelista A, Del Bianco A, Giordano G, Brisciani M, Piazzolla M, Zullino V, Tozzi P, Galardo G, De Giacomo T, Ruberto F (2022). Non-intubated thoracic Surgery: standpoints and perspectives. Front Surg.

