# Supplementary material for: Airway management for a patient with tracheobronchomegaly undergoing lobectomy: a case report
Source: BMC Anesthesiol. 2023 Nov 2;23:357. doi: 10.1186/s12871-023-02324-5 (PMC10621132; doi:10.1186/s12871-023-02324-5)

**Figure Legends**

**Figure:** Occluding the proximal opening of the modified DFC’ drainage lumen

a. To make it easy, tying a suture to the sealing cap of a 20 G BD arterial cannula (arrow “B”), the arrow “A” represented the proximal opening of the modified DFC’ drainage lumen. b. Occluding the proximal opening of the modified DFC’ drainage lumen (arrow “A”) with the help of surgical forceps. c. Status after occluding.


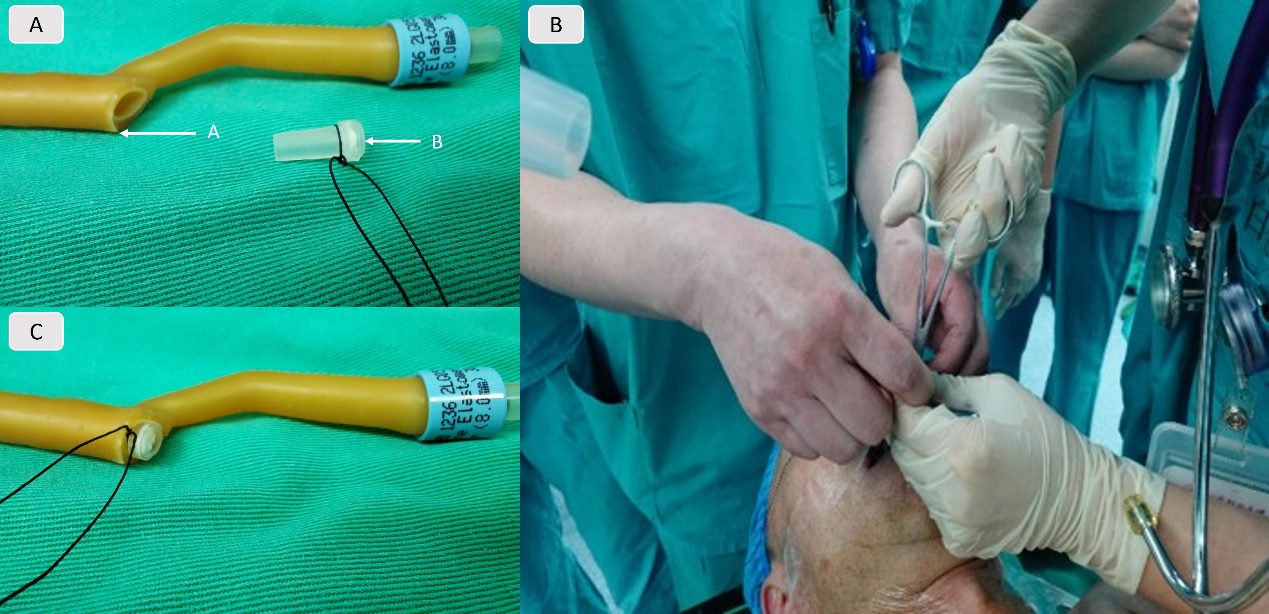

Supplement: Supplementary file 1 — Supplementary Material 1 [file 12871_2023_2324_MOESM1_ESM.docx]
